# Chlorido{5,5′-dimethyl-2,2′-[1,2-phenyl­enebis(nitrilo­methyl­idyne)]diphenolato-κ^4^ *O*,*N*,*N*′,*O*′}manganese(III)

**DOI:** 10.1107/S1600536808007459

**Published:** 2008-03-29

**Authors:** Naser Eltaher Eltayeb, Siang Guan Teoh, Suchada Chantrapromma, Hoong-Kun Fun, Rohana Adnan

**Affiliations:** a School of Chemical Science, Universiti Sains Malaysia, 11800 USM, Penang, Malaysia; b Department of Chemistry, Faculty of Science, Prince of Songkla University, Hat-Yai, Songkhla 90112, Thailand; c X-ray Crystallography Unit, School of Physics, Universiti Sains Malaysia, 11800 USM, Penang, Malaysia

## Abstract

In the title complex, [Mn(C_22_H_18_N_2_O_2_)Cl], the Mn^III^ center is in a distorted square-pyramidal configuration; the basal plane is formed by the N_2_O_2_ donors of the tetra­dentate Schiff base dianion, with the two phenol O atoms and two imine N atoms each mutually *cis*. The chloride ion occupies the apical coordination site. The dihedral angle between the two outer phenolate rings of the tetra­dentate ligand is 18.24 (9)°. The central benzene ring makes dihedral angles of 13.71 (8) and 30.50 (8)° with the two outer phenolate rings. In the crystal structure, weak C—H⋯Cl inter­actions link the mol­ecules into screw helices along the *b* direction. These helices are further connected by weak C—H⋯O inter­actions into a three-dimensional network. The crystal structure is further stabilized by C—H⋯π inter­actions involving the central benzene ring.

## Related literature

For values of bond lengths, see: Allen *et al.* (1987 ▶). For details of ring conformations, see: Cremer & Pople (1975 ▶). For related structures, see, for example: Eltayeb *et al.* (2008 ▶); Habibi *et al.* (2007 ▶); Mitra *et al.* (2006 ▶). For background to applications of manganese complexes, see, for example: Dixit & Srinivasan (1988 ▶); Glatzel *et al.* (2004 ▶); Lu *et al.* (2006 ▶); Stallings *et al.* (1985 ▶).
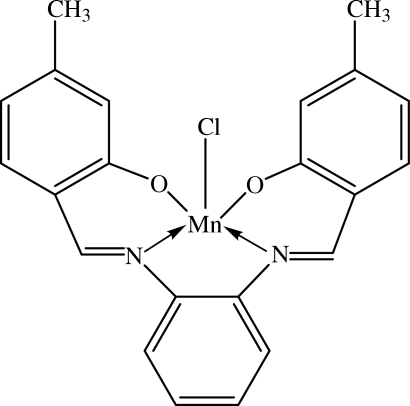


## Experimental

### 

#### Crystal data

[Mn(C_22_H_18_N_2_O_2_)Cl]
*M* *_r_* = 432.77
Monoclinic, 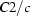

*a* = 20.9593 (5) Å
*b* = 13.5897 (3) Å
*c* = 14.9316 (3) Å
β = 119.641 (1)°
*V* = 3696.43 (14) Å^3^
*Z* = 8
Mo *K*α radiation
μ = 0.88 mm^−1^
*T* = 100.0 (1) K
0.56 × 0.20 × 0.19 mm

#### Data collection

Bruker SMART APEXII CCD area-detector diffractometer
Absorption correction: multi-scan (*SADABS*; Bruker, 2005 ▶) *T*_min_ = 0.639, *T*_max_ = 0.852
35769 measured reflections
8109 independent reflections
5992 reflections with *I* > 2σ(*I*)
*R*_int_ = 0.048

#### Refinement

*R*[*F* ^2^ > 2σ(*F* ^2^)] = 0.045
*wR*(*F* ^2^) = 0.126
*S* = 1.07
8109 reflections
255 parameters
H-atom parameters constrained
Δρ_max_ = 0.75 e Å^−3^
Δρ_min_ = −0.69 e Å^−3^

### 

Data collection: *APEX2* (Bruker, 2005 ▶); cell refinement: *APEX2*; data reduction: *SAINT* (Bruker, 2005 ▶); program(s) used to solve structure: *SHELXTL* (Sheldrick, 2008 ▶); program(s) used to refine structure: *SHELXTL*; molecular graphics: *SHELXTL*; software used to prepare material for publication: *SHELXTL* and *PLATON* (Spek, 2003 ▶).

## Supplementary Material

Crystal structure: contains datablocks global, I. DOI: 10.1107/S1600536808007459/sj2474sup1.cif

Structure factors: contains datablocks I. DOI: 10.1107/S1600536808007459/sj2474Isup2.hkl

Additional supplementary materials:  crystallographic information; 3D view; checkCIF report

## Figures and Tables

**Table 1:** Hydrogen-bond geometry (Å, °)

*D*—H⋯*A*	*D*—H	H⋯*A*	*D*⋯*A*	*D*—H⋯*A*
C5—H5*A*⋯Cl1^i^	0.93	2.77	3.6508 (16)	158
C7—H7*A*⋯Cl1^i^	0.93	2.81	3.6933 (15)	158
C11—H11*A*⋯O1^ii^	0.93	2.58	3.423 (2)	151
C4—H4*A*⋯*Cg*1^iii^	0.93	2.83	3.5443 (19)	135

Symmetry codes: (i) 
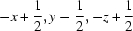
; (ii) 
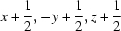
; (iii) 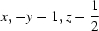. *Cg*1 is the centroid of the C8–C13 benzene ring.

**Fig. 1.**
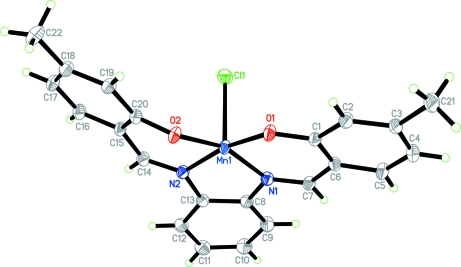
The asymmetric unit of (I), showing 50% probability displacement ellipsoids and the atomic numbering.

**Fig. 2.**
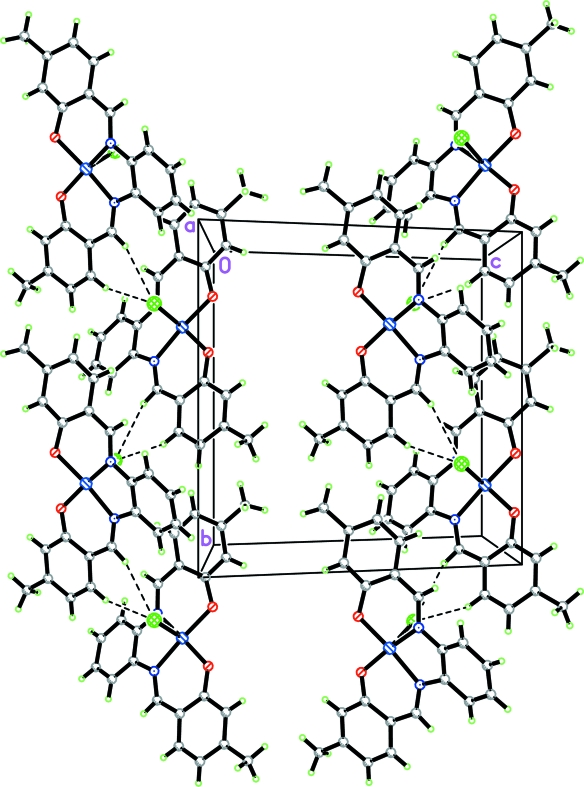
The crystal packing of (I), viewed along the a axis, showing the three-dimensional network. C—H···Cl weak interactions are drawn as dashed lines.

## supplementary crystallographic information

### Comment

We have been interested in syntheses of Schiff base ligands containing oxygen and imine nitrogen atoms and their metal complexes due to their variety of applications. Manganese complexes with Schiff base ligands have numerous applications in chemistry, biology, physics and advanced materials and are used in catalysis (Dixit and Srinivasan, 1988), as models for the oxygen-evolving complex of photosystem II (Glatzel *et al.*, 2004), and as single-molecule magnets (Lu *et al.*, 2006). They also serve as models for the active sites of manganese-containing metal enzymes (Stallings *et al.*, 1985).  Recently, we reported the crystal structure of  a five coordinate Mn^III^ complex with a similar N_2_O_2_ donor Schiff base ligand, chlorido{6,6'-dimethyl-2,2'-[1,2-phenylenebis(nitrilomethylidene)]diphenolato- κ^4^*O*,*N*,*N*',*O*'}manganese(III) monohydrate (Eltayeb *et al.*, 2008). We report here the structure of (I), Fig. 1, a Mn^III^ complex of a closely-related ligand.

In (I) the Mn^III^ center is in a slightly distorted square-pyramidal geometry coordinating through N1, N2, O1 and O2 atoms of the tetradentate Schiff base ligand in the basal plane with the two phenolic O atoms and two imine N atoms in mutually *cis* positions.  The apical position is coordinated by the Cl^-^ ion. The Mn—O distances [Mn1—O1 = 1.8698 (12)Å, Mn1—O2 = 1.8983 (10)Å] and Mn—N distances [Mn1—N1 = 1.9923 (12)Å, Mn1—N2 = 1.9875 (12)Å]  are in the same ranges of those observed in other related Mn^III^ complexes of N_2_O_2_ Schiff base ligands (Eltayeb *et al.*, 2008; Habibi *et al.*, 2007; Mitra *et al.*, 2006). Other bond lengths and angles observed in the structure are also normal (Allen *et al.*, 1987). The basal bond angles O1–Mn1–O2 of 92.61 (4)°, O–Mn–N [O1–Mn1–N1 = 93.07 (5)°, O2–Mn1–N2 = 89.21 (5)°] are close to 90° whereas the N–Mn–N is smaller than 90° [N1–Mn1–N2 = 82.10 (5)°]. The bond angles between the Cl^-^ ion and the atoms in the basal plane are in the range 93.14 (4) to 99.97 (4)°, indicating a distorted square-pyramidal geometry. Coordination of the the N_2_O_2_ chelate ligand to the Mn^III^ ion results in the formation of an essentialy planar five-membered ring (Mn1/N1/N2/C8/C13) and two six-membered rings; the Mn1/O1/N1/C1/C6/C7 ring is almost planar with the greatest deviation being 0.059 (1)Å for atom O2 whereas the Mn1/O2/N2/C14/C15/C20 ring adopts an envelope conformation with atom O2 displaced from the Mn1/N2/C14/C15/C20 plane by 0.298 (1)Å and with Cremer & Pople (1975) puckering parameters Q = 0.483 (1)°, θ = 61.0 (1)° and φ = 18.8 (2)°. These parameters are larger in values than those observed in the closely-related structure (Eltayeb *et al.*, 2008). The dihedral angle between the two outer phenolate rings [C1–C6 and C15–C20] of the Schiff base ligand is 18.24 (9)°. The central benzene ring (C8–C13) makes dihedral angles of 13.71 (8)° and 30.50 (8)° with the two outer phenolate rings. These dihedral angles are all wider than the corresponding angles found in a closely related structure (Eltayeb *et al.*, 2008) due to the different locations of the two methyl substituents on the phenolate rings of the Schiff base ligand.

In the crystal packing (Fig. 2), weak C—H···Cl interactions (Table 1) link the molecules into screw helices along the *b* direction. These helices are further connected by weak C—H···O interactions into a three-dimensional network. The crystal is further stabilized by weak C—H···π interactions (Table 1); *Cg*_1_ is the centroid of the C8–C13 benzene ring.

### Experimental

The title compound was synthesized by adding 2-hydroxy-4-methylbenzaldehyde (0.546 g, 4 mmol) to a solution of *o*-phenylenediamine (0.216 g, 2 mmol) in ethanol 95% (30 ml). The mixture was refluxed with stirring for half an hour. Manganese chloride tetrahydrate (0.394 g, 2 mmol) in ethanol (10 ml) was then added, followed by triethylamine (0.5 ml, 3.6 mmol). The mixture was refluxed at room temperature for three hours. A brown precipitate was obtained, washed with about 5 ml ethanol, dried, and then washed with copious quantities of diethylether. Brown single crystals of the title compound suitable for *x*-ray structure determination were recrystallized from ethanol/methanol (2:1 *v*/*v*) by slow evaporation of the solvent at room temperature over two months.

### Refinement

All H atoms were placed in calculated positions with d(C—H) = 0.93 Å, *U*_iso_=1.2*U*_eq_(C) for aromatic and CH, 0.96 Å, *U*_iso_ = 1.5*U*_eq_(C) for CH_3_ atoms. A rotating group model was used for the methyl groups. The highest residual electron density peak is located at 0.81 Å from Cl1 and the deepest hole is located at 0.67 Å from Mn1.

### Crystal data

**Table:**

[Mn(C_22_H_18_N_2_O_2_)Cl]	*F*_000_ = 1776
*M**_r_* = 432.77	*D*_x_ = 1.555 Mg m^−^^3^
Monoclinic, *C*2/*c*	Mo *K*α radiation λ = 0.71073 Å
Hall symbol: -C 2yc	Cell parameters from 8109 reflections
*a* = 20.9593 (5) Å	θ = 2.1–35.0º
*b* = 13.5897 (3) Å	µ = 0.88 mm^−^^1^
*c* = 14.9316 (3) Å	*T* = 100.0 (1) K
β = 119.641 (1)º	Block, brown
*V* = 3696.43 (14) Å^3^	0.56 × 0.20 × 0.19 mm
*Z* = 8	

### Data collection

**Table:**

Bruker SMART APEXII CCD area-detector diffractometer	8109 independent reflections
Radiation source: fine-focus sealed tube	5992 reflections with *I* > 2σ(*I*)
Monochromator: graphite	*R*_int_ = 0.048
Detector resolution: 8.33 pixels mm^-1^	θ_max_ = 35.0º
*T* = 100.0(1) K	θ_min_ = 2.1º
ω scans	*h* = −33→28
Absorption correction: multi-scan(SADABS; Bruker, 2005)	*k* = −21→21
*T*_min_ = 0.639, *T*_max_ = 0.852	*l* = −23→24
35769 measured reflections	

### Refinement

**Table:**

Refinement on *F*^2^	Secondary atom site location: difference Fourier map
Least-squares matrix: full	Hydrogen site location: inferred from neighbouring sites
*R*[*F*^2^ > 2σ(*F*^2^)] = 0.045	H-atom parameters constrained
*wR*(*F*^2^) = 0.126	*w* = 1/[σ^2^(*F*_o_^2^) + (0.0635*P*)^2^ + 1.3131*P*] where *P* = (*F*_o_^2^ + 2*F*_c_^2^)/3
*S* = 1.07	(Δ/σ)_max_ = 0.001
8109 reflections	Δρ_max_ = 0.75 e Å^−^^3^
255 parameters	Δρ_min_ = −0.69 e Å^−^^3^
Primary atom site location: structure-invariant direct methods	Extinction correction: none

### Special details

**Table:**

Experimental. The low-temperature data was collected with the Oxford Cyrosystem Cobra low-temperature attachment.
Geometry. All e.s.d.'s (except the e.s.d. in the dihedral angle between two l.s. planes) are estimated using the full covariance matrix. The cell e.s.d.'s are taken into account individually in the estimation of e.s.d.'s in distances, angles and torsion angles; correlations between e.s.d.'s in cell parameters are only used when they are defined by crystal symmetry. An approximate (isotropic) treatment of cell e.s.d.'s is used for estimating e.s.d.'s involving l.s. planes.
Refinement. Refinement of *F*^2^ against ALL reflections. The weighted *R*-factor *wR* and goodness of fit *S* are based on *F*^2^, conventional *R*-factors *R* are based on *F*, with *F* set to zero for negative *F*^2^. The threshold expression of *F*^2^ > σ(*F*^2^) is used only for calculating *R*-factors(gt) *etc*. and is not relevant to the choice of reflections for refinement. *R*-factors based on *F*^2^ are statistically about twice as large as those based on *F*, and *R*- factors based on ALL data will be even larger.

### Fractional atomic coordinates and isotropic or equivalent isotropic displacement parameters (Å^2^)

**Table:**

	*x*	*y*	*z*	*U*_iso_*/*U*_eq_	
Mn1	0.225261 (11)	0.228215 (15)	0.091917 (16)	0.01606 (6)	
Cl1	0.16386 (2)	0.29835 (3)	0.17860 (3)	0.02432 (9)	
O1	0.14852 (6)	0.14593 (7)	0.00249 (8)	0.0192 (2)	
O2	0.19870 (6)	0.32753 (7)	−0.00984 (8)	0.0185 (2)	
N1	0.27984 (6)	0.11876 (9)	0.18852 (9)	0.0168 (2)	
N2	0.31816 (6)	0.30016 (9)	0.18058 (9)	0.0167 (2)	
C1	0.13609 (8)	0.05345 (10)	0.01522 (11)	0.0174 (3)	
C2	0.07011 (8)	0.00997 (11)	−0.05872 (12)	0.0219 (3)	
H2A	0.0365	0.0481	−0.1136	0.026*	
C3	0.05302 (8)	−0.08722 (11)	−0.05333 (12)	0.0209 (3)	
C4	0.10445 (9)	−0.14629 (12)	0.02727 (12)	0.0226 (3)	
H4A	0.0941	−0.2121	0.0314	0.027*	
C5	0.17011 (8)	−0.10657 (11)	0.10001 (11)	0.0209 (3)	
H5A	0.2043	−0.1468	0.1520	0.025*	
C6	0.18698 (8)	−0.00631 (10)	0.09789 (10)	0.0168 (2)	
C7	0.25658 (8)	0.02818 (11)	0.17669 (10)	0.0173 (2)	
H7A	0.2881	−0.0180	0.2237	0.021*	
C8	0.35313 (7)	0.14389 (11)	0.26289 (10)	0.0177 (3)	
C9	0.40356 (8)	0.08078 (12)	0.33913 (12)	0.0233 (3)	
H9A	0.3889	0.0190	0.3489	0.028*	
C10	0.47580 (8)	0.11154 (13)	0.40001 (12)	0.0263 (3)	
H10A	0.5094	0.0701	0.4512	0.032*	
C11	0.49860 (8)	0.20291 (13)	0.38569 (12)	0.0258 (3)	
H11A	0.5475	0.2215	0.4251	0.031*	
C12	0.44804 (8)	0.26669 (12)	0.31221 (12)	0.0229 (3)	
H12A	0.4631	0.3282	0.3024	0.027*	
C13	0.37498 (8)	0.23870 (11)	0.25325 (11)	0.0174 (3)	
C14	0.32367 (8)	0.39542 (11)	0.17937 (11)	0.0193 (3)	
H14A	0.3669	0.4242	0.2297	0.023*	
C15	0.26777 (8)	0.45912 (10)	0.10593 (11)	0.0179 (3)	
C16	0.27519 (9)	0.56198 (11)	0.12163 (12)	0.0216 (3)	
H16A	0.3150	0.5867	0.1811	0.026*	
C17	0.22469 (9)	0.62645 (11)	0.05081 (12)	0.0235 (3)	
H17A	0.2306	0.6938	0.0630	0.028*	
C18	0.16426 (9)	0.59053 (11)	−0.03985 (12)	0.0216 (3)	
C19	0.15624 (8)	0.48953 (11)	−0.05583 (11)	0.0196 (3)	
H19A	0.1159	0.4657	−0.1152	0.024*	
C20	0.20661 (8)	0.42298 (10)	0.01426 (11)	0.0176 (3)	
C21	−0.01947 (9)	−0.12957 (13)	−0.13236 (15)	0.0305 (4)	
H21A	−0.0443	−0.0835	−0.1878	0.046*	
H21B	−0.0491	−0.1423	−0.1011	0.046*	
H21C	−0.0114	−0.1899	−0.1587	0.046*	
C22	0.10955 (10)	0.65886 (13)	−0.12034 (14)	0.0305 (4)	
H22A	0.1032	0.6414	−0.1866	0.046*	
H22B	0.1271	0.7253	−0.1040	0.046*	
H22C	0.0634	0.6535	−0.1218	0.046*	

### Atomic displacement parameters (Å^2^)

**Table:**

	*U*^11^	*U*^22^	*U*^33^	*U*^12^	*U*^13^	*U*^23^
Mn1	0.01366 (10)	0.01200 (11)	0.01705 (10)	−0.00147 (7)	0.00341 (7)	0.00028 (7)
Cl1	0.02102 (17)	0.02319 (18)	0.02899 (18)	−0.00435 (13)	0.01255 (14)	−0.00675 (13)
O1	0.0161 (5)	0.0125 (4)	0.0212 (5)	−0.0013 (4)	0.0033 (4)	0.0006 (3)
O2	0.0210 (5)	0.0112 (4)	0.0180 (4)	−0.0003 (4)	0.0057 (4)	−0.0002 (3)
N1	0.0142 (5)	0.0162 (5)	0.0164 (5)	−0.0010 (4)	0.0049 (4)	0.0001 (4)
N2	0.0142 (5)	0.0148 (5)	0.0183 (5)	−0.0012 (4)	0.0058 (4)	−0.0014 (4)
C1	0.0150 (6)	0.0136 (6)	0.0218 (6)	−0.0009 (5)	0.0077 (5)	−0.0011 (5)
C2	0.0139 (6)	0.0168 (7)	0.0271 (7)	−0.0012 (5)	0.0041 (5)	−0.0017 (5)
C3	0.0149 (6)	0.0174 (7)	0.0280 (7)	−0.0028 (5)	0.0087 (5)	−0.0033 (5)
C4	0.0251 (7)	0.0174 (7)	0.0250 (7)	−0.0053 (6)	0.0121 (6)	−0.0018 (5)
C5	0.0231 (7)	0.0147 (6)	0.0214 (6)	−0.0012 (5)	0.0084 (5)	0.0013 (5)
C6	0.0153 (6)	0.0146 (6)	0.0188 (6)	−0.0016 (5)	0.0070 (5)	0.0000 (4)
C7	0.0175 (6)	0.0154 (6)	0.0170 (5)	0.0002 (5)	0.0070 (5)	0.0012 (4)
C8	0.0133 (6)	0.0181 (6)	0.0180 (6)	0.0000 (5)	0.0049 (5)	−0.0002 (5)
C9	0.0166 (6)	0.0227 (7)	0.0230 (6)	0.0002 (5)	0.0041 (5)	0.0039 (5)
C10	0.0166 (7)	0.0282 (8)	0.0242 (7)	0.0023 (6)	0.0025 (5)	0.0038 (6)
C11	0.0142 (6)	0.0295 (8)	0.0264 (7)	−0.0010 (6)	0.0044 (5)	−0.0017 (6)
C12	0.0149 (6)	0.0217 (7)	0.0272 (7)	−0.0028 (5)	0.0067 (5)	−0.0032 (5)
C13	0.0144 (6)	0.0171 (6)	0.0178 (6)	−0.0011 (5)	0.0057 (5)	−0.0022 (5)
C14	0.0181 (6)	0.0172 (6)	0.0205 (6)	−0.0037 (5)	0.0080 (5)	−0.0031 (5)
C15	0.0171 (6)	0.0129 (6)	0.0223 (6)	−0.0023 (5)	0.0087 (5)	−0.0011 (5)
C16	0.0233 (7)	0.0155 (6)	0.0253 (7)	−0.0033 (5)	0.0115 (6)	−0.0026 (5)
C17	0.0280 (8)	0.0132 (6)	0.0308 (7)	−0.0001 (5)	0.0158 (6)	−0.0011 (5)
C18	0.0250 (7)	0.0171 (7)	0.0247 (7)	0.0029 (5)	0.0139 (6)	0.0022 (5)
C19	0.0210 (7)	0.0163 (6)	0.0212 (6)	0.0012 (5)	0.0102 (5)	0.0003 (5)
C20	0.0191 (6)	0.0138 (6)	0.0201 (6)	−0.0016 (5)	0.0097 (5)	−0.0006 (5)
C21	0.0176 (7)	0.0219 (8)	0.0417 (9)	−0.0054 (6)	0.0068 (6)	−0.0063 (7)
C22	0.0371 (9)	0.0193 (7)	0.0317 (8)	0.0082 (7)	0.0143 (7)	0.0026 (6)

### Geometric parameters (Å, °)

**Table:**

Mn1—O1	1.8698 (10)	C9—H9A	0.9300
Mn1—O2	1.8983 (10)	C10—C11	1.385 (2)
Mn1—N2	1.9875 (12)	C10—H10A	0.9300
Mn1—N1	1.9923 (12)	C11—C12	1.388 (2)
Mn1—Cl1	2.4263 (4)	C11—H11A	0.9300
O1—C1	1.3162 (17)	C12—C13	1.390 (2)
O2—C20	1.3344 (17)	C12—H12A	0.9300
N1—C7	1.3034 (18)	C14—C15	1.433 (2)
N1—C8	1.4221 (17)	C14—H14A	0.9300
N2—C14	1.3006 (19)	C15—C16	1.413 (2)
N2—C13	1.4195 (18)	C15—C20	1.4221 (19)
C1—C2	1.4036 (19)	C16—C17	1.378 (2)
C1—C6	1.4208 (19)	C16—H16A	0.9300
C2—C3	1.381 (2)	C17—C18	1.407 (2)
C2—H2A	0.9300	C17—H17A	0.9300
C3—C4	1.404 (2)	C18—C19	1.389 (2)
C3—C21	1.503 (2)	C18—C22	1.503 (2)
C4—C5	1.373 (2)	C19—C20	1.391 (2)
C4—H4A	0.9300	C19—H19A	0.9300
C5—C6	1.412 (2)	C21—H21A	0.9600
C5—H5A	0.9300	C21—H21B	0.9600
C6—C7	1.4263 (19)	C21—H21C	0.9600
C7—H7A	0.9300	C22—H22A	0.9600
C8—C13	1.398 (2)	C22—H22B	0.9600
C8—C9	1.399 (2)	C22—H22C	0.9600
C9—C10	1.390 (2)		
			
O1—Mn1—O2	92.61 (4)	C11—C10—C9	121.08 (14)
O1—Mn1—N2	169.49 (5)	C11—C10—H10A	119.5
O2—Mn1—N2	89.21 (5)	C9—C10—H10A	119.5
O1—Mn1—N1	93.07 (5)	C10—C11—C12	119.67 (14)
O2—Mn1—N1	161.15 (5)	C10—C11—H11A	120.2
N2—Mn1—N1	82.10 (5)	C12—C11—H11A	120.2
O1—Mn1—Cl1	96.89 (4)	C11—C12—C13	120.06 (15)
O2—Mn1—Cl1	97.16 (4)	C11—C12—H12A	120.0
N2—Mn1—Cl1	93.14 (4)	C13—C12—H12A	120.0
N1—Mn1—Cl1	99.97 (4)	C12—C13—C8	120.09 (13)
C1—O1—Mn1	129.03 (9)	C12—C13—N2	124.62 (14)
C20—O2—Mn1	121.82 (9)	C8—C13—N2	115.29 (12)
C7—N1—C8	121.32 (12)	N2—C14—C15	124.56 (13)
C7—N1—Mn1	124.38 (10)	N2—C14—H14A	117.7
C8—N1—Mn1	113.44 (9)	C15—C14—H14A	117.7
C14—N2—C13	123.34 (12)	C16—C15—C20	118.30 (13)
C14—N2—Mn1	123.02 (10)	C16—C15—C14	119.30 (13)
C13—N2—Mn1	113.25 (9)	C20—C15—C14	122.31 (13)
O1—C1—C2	118.48 (13)	C17—C16—C15	121.46 (14)
O1—C1—C6	123.65 (12)	C17—C16—H16A	119.3
C2—C1—C6	117.84 (13)	C15—C16—H16A	119.3
C3—C2—C1	122.67 (14)	C16—C17—C18	120.13 (14)
C3—C2—H2A	118.7	C16—C17—H17A	119.9
C1—C2—H2A	118.7	C18—C17—H17A	119.9
C2—C3—C4	119.04 (13)	C19—C18—C17	118.89 (14)
C2—C3—C21	120.74 (14)	C19—C18—C22	119.57 (14)
C4—C3—C21	120.23 (14)	C17—C18—C22	121.52 (14)
C5—C4—C3	119.82 (14)	C18—C19—C20	122.06 (14)
C5—C4—H4A	120.1	C18—C19—H19A	119.0
C3—C4—H4A	120.1	C20—C19—H19A	119.0
C4—C5—C6	121.72 (14)	O2—C20—C19	118.83 (13)
C4—C5—H5A	119.1	O2—C20—C15	121.86 (13)
C6—C5—H5A	119.1	C19—C20—C15	119.15 (13)
C5—C6—C1	118.84 (12)	C3—C21—H21A	109.5
C5—C6—C7	117.65 (13)	C3—C21—H21B	109.5
C1—C6—C7	123.43 (13)	H21A—C21—H21B	109.5
N1—C7—C6	125.70 (13)	C3—C21—H21C	109.5
N1—C7—H7A	117.2	H21A—C21—H21C	109.5
C6—C7—H7A	117.2	H21B—C21—H21C	109.5
C13—C8—C9	119.63 (13)	C18—C22—H22A	109.5
C13—C8—N1	115.03 (12)	C18—C22—H22B	109.5
C9—C8—N1	125.29 (13)	H22A—C22—H22B	109.5
C10—C9—C8	119.20 (15)	C18—C22—H22C	109.5
C10—C9—H9A	120.4	H22A—C22—H22C	109.5
C8—C9—H9A	120.4	H22B—C22—H22C	109.5
			
O2—Mn1—O1—C1	170.43 (13)	C5—C6—C7—N1	−177.31 (15)
N2—Mn1—O1—C1	70.7 (3)	C1—C6—C7—N1	6.2 (2)
N1—Mn1—O1—C1	8.41 (13)	C7—N1—C8—C13	167.53 (14)
Cl1—Mn1—O1—C1	−92.03 (12)	Mn1—N1—C8—C13	−2.25 (16)
O1—Mn1—O2—C20	145.34 (11)	C7—N1—C8—C9	−9.7 (2)
N2—Mn1—O2—C20	−45.01 (11)	Mn1—N1—C8—C9	−179.47 (13)
N1—Mn1—O2—C20	−107.23 (17)	C13—C8—C9—C10	−3.8 (2)
Cl1—Mn1—O2—C20	48.06 (11)	N1—C8—C9—C10	173.30 (15)
O1—Mn1—N1—C7	−1.63 (13)	C8—C9—C10—C11	−0.6 (3)
O2—Mn1—N1—C7	−108.98 (16)	C9—C10—C11—C12	2.6 (3)
N2—Mn1—N1—C7	−172.25 (13)	C10—C11—C12—C13	−0.1 (3)
Cl1—Mn1—N1—C7	95.93 (12)	C11—C12—C13—C8	−4.3 (2)
O1—Mn1—N1—C8	167.79 (10)	C11—C12—C13—N2	176.11 (15)
O2—Mn1—N1—C8	60.44 (19)	C9—C8—C13—C12	6.3 (2)
N2—Mn1—N1—C8	−2.83 (10)	N1—C8—C13—C12	−171.13 (14)
Cl1—Mn1—N1—C8	−94.65 (10)	C9—C8—C13—N2	−174.11 (13)
O1—Mn1—N2—C14	131.1 (2)	N1—C8—C13—N2	8.51 (19)
O2—Mn1—N2—C14	31.04 (12)	C14—N2—C13—C12	−18.1 (2)
N1—Mn1—N2—C14	−165.73 (13)	Mn1—N2—C13—C12	168.88 (13)
Cl1—Mn1—N2—C14	−66.09 (12)	C14—N2—C13—C8	162.28 (14)
O1—Mn1—N2—C13	−55.8 (3)	Mn1—N2—C13—C8	−10.73 (16)
O2—Mn1—N2—C13	−155.92 (10)	C13—N2—C14—C15	178.88 (14)
N1—Mn1—N2—C13	7.31 (10)	Mn1—N2—C14—C15	−8.8 (2)
Cl1—Mn1—N2—C13	106.95 (10)	N2—C14—C15—C16	170.29 (15)
Mn1—O1—C1—C2	173.26 (11)	N2—C14—C15—C20	−13.2 (2)
Mn1—O1—C1—C6	−9.1 (2)	C20—C15—C16—C17	0.3 (2)
O1—C1—C2—C3	178.35 (15)	C14—C15—C16—C17	176.93 (15)
C6—C1—C2—C3	0.6 (2)	C15—C16—C17—C18	−0.3 (2)
C1—C2—C3—C4	−2.1 (2)	C16—C17—C18—C19	0.6 (2)
C1—C2—C3—C21	177.86 (16)	C16—C17—C18—C22	−177.92 (16)
C2—C3—C4—C5	1.0 (2)	C17—C18—C19—C20	−0.9 (2)
C21—C3—C4—C5	−179.03 (16)	C22—C18—C19—C20	177.67 (15)
C3—C4—C5—C6	1.7 (2)	Mn1—O2—C20—C19	−146.85 (11)
C4—C5—C6—C1	−3.3 (2)	Mn1—O2—C20—C15	37.70 (18)
C4—C5—C6—C7	−179.96 (15)	C18—C19—C20—O2	−174.69 (14)
O1—C1—C6—C5	−175.56 (14)	C18—C19—C20—C15	0.9 (2)
C2—C1—C6—C5	2.1 (2)	C16—C15—C20—O2	174.89 (14)
O1—C1—C6—C7	0.9 (2)	C14—C15—C20—O2	−1.6 (2)
C2—C1—C6—C7	178.55 (14)	C16—C15—C20—C19	−0.6 (2)
C8—N1—C7—C6	−173.22 (14)	C14—C15—C20—C19	−177.07 (14)
Mn1—N1—C7—C6	−4.6 (2)		

### Hydrogen-bond geometry (Å, °)

**Table:**

*D*—H···*A*	*D*—H	H···*A*	*D*···*A*	*D*—H···*A*
C5—H5A···Cl1^i^	0.93	2.77	3.6508 (16)	158
C7—H7A···Cl1^i^	0.93	2.81	3.6933 (15)	158
C11—H11A···O1^ii^	0.93	2.58	3.423 (2)	151
C4—H4A···Cg1^iii^	0.93	2.83	3.5443 (19)	135

Symmetry codes: (i) −*x*+1/2, *y*−1/2, −*z*+1/2; (ii) *x*+1/2, −*y*+1/2, *z*+1/2; (iii) *x*, −*y*−1, *z*−1/2.
